# Supplementary material for: Engineering N-acyl-homoserine lactone-based quorum-sensing circuit for dynamic regulatory control in Saccharomyces cerevisiae
Source: Commun Biol. 2025 Nov 27;8:1793. doi: 10.1038/s42003-025-09163-9 (PMC12717128; doi:10.1038/s42003-025-09163-9)
Supplement: Supplementary file 1 — Supplementary information [file 42003_2025_9163_MOESM1_ESM.pdf]

Supplementary Information

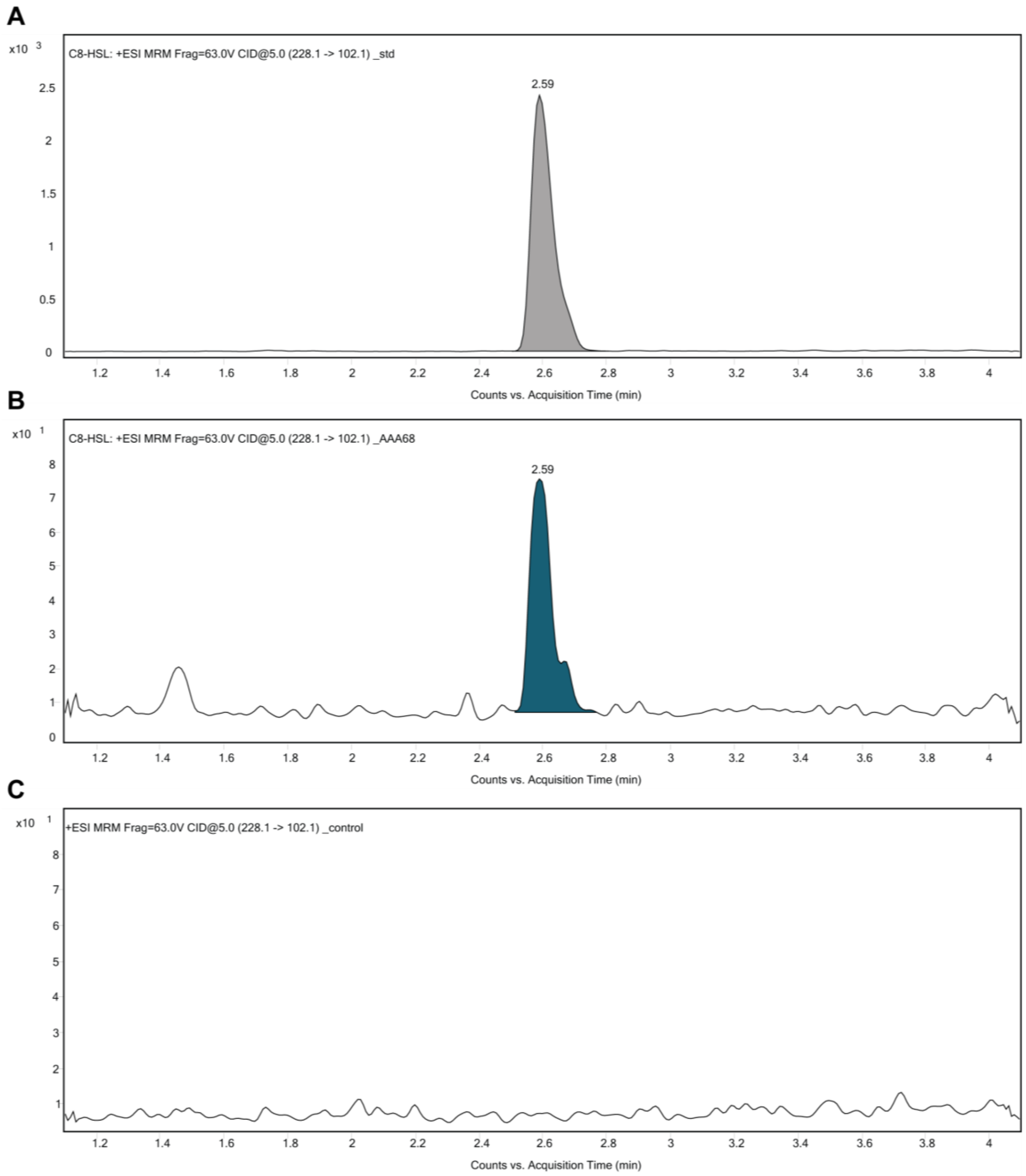

Figure S1: Production of C8-HSL established in yeast. (A) LC-MS chromatogram of a standard of C8-HSL. LC MS chromatogram of supernatant of (B) *S. cerevisiae* strain AAA068 (*Bc.cepI*) and (C) CEN.PK2-1C as control, grown for 24 hours on synthetic complete (SC) medium. C8-HSL eluted at a retention time of 2.6 min. Representative data of biological duplicates and 3 technical replicates of the standard are shown.

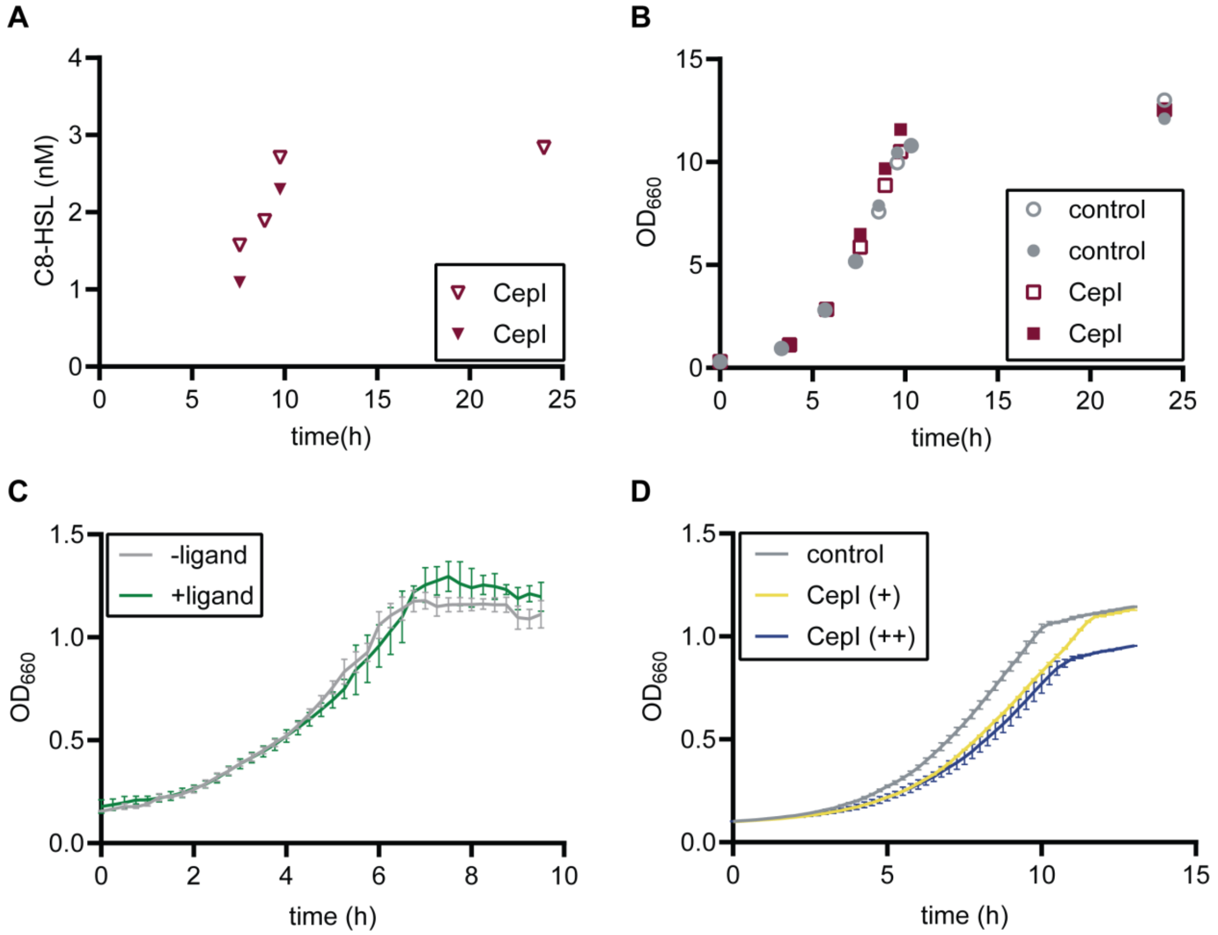

Figure S2: Growth and C8-HSL production of engineered yeast strains. (A) Duplicate cultures of *S. cerevisiae* strains CEN.PK2-1C (control) and AAA068 (CepI), were grown in aerobic shakeflasks on synthetic complete (SC) medium at 30°C and growth was analysed by manual OD_{600} measurements and (B) concentrations of C8-HSL was determined by LC-MS for strain AAA068. (C) Triplicate cultures of CEN.PK2-1C were grown on SC medium with and without supplementation of 100 µM of C8-HSL and growth was monitored by OD_{660} measurements in a plate reader. (D) Triplicate cultures of strains CEN.PK2-1C (control), AAA113 (CepI (+)) and AAA111 (CepI (++)) were grown on fed-batch medium with supplementation of methionine and growth was monitored by OD_{660} measurements in a plate reader.

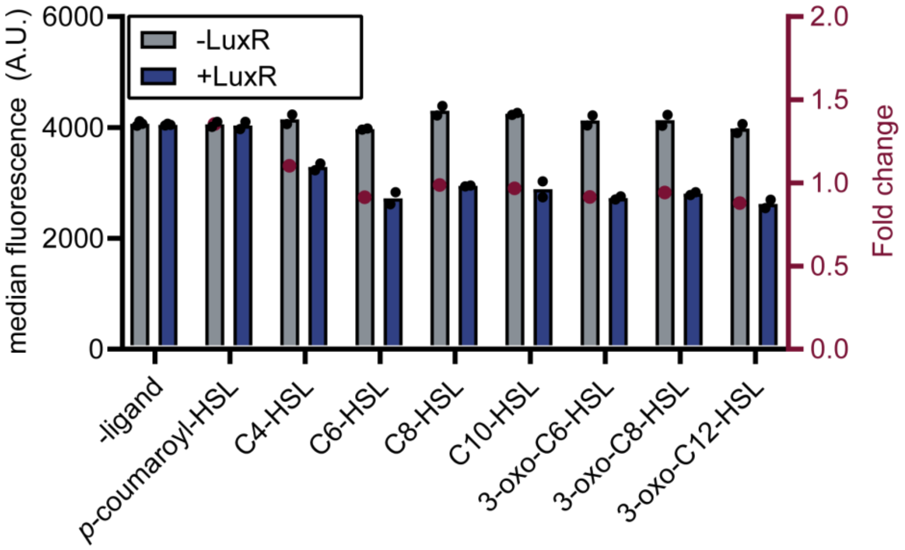

Figure S3: Fluorescent response of LuxR equipped without activation domain to different AHLs. Bars indicate yeGFP fluorescence levels 6 h following supplementation of 0 or 100 μM ligand tested for strains AAA019 (-LuxR) and AAA035 (+LuxR) in duplicate cultures, measured by flow cytometer. Red dots indicate fold change in yeGFP fluorescence between 0 μM and 100 μM treatment.

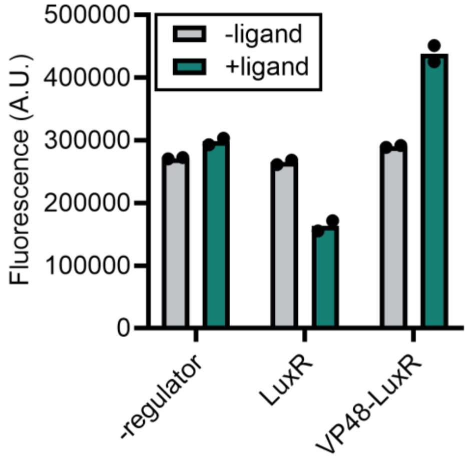

Figure S4: Fluorescent response to C8-HSL of LuxR biosensors equipped with and without activation domain and with the operator sequence positioned downstream of the TATA-box. Bars indicate yeGFP fluorescence levels 6 h following supplementation of 0 or 100 μM C8-HSL tested for strains AAA014 (-regulator), AAA071 (LuxR) and AAA070 (VP48-LuxR) in duplicate cultures, measured by flow cytometer.

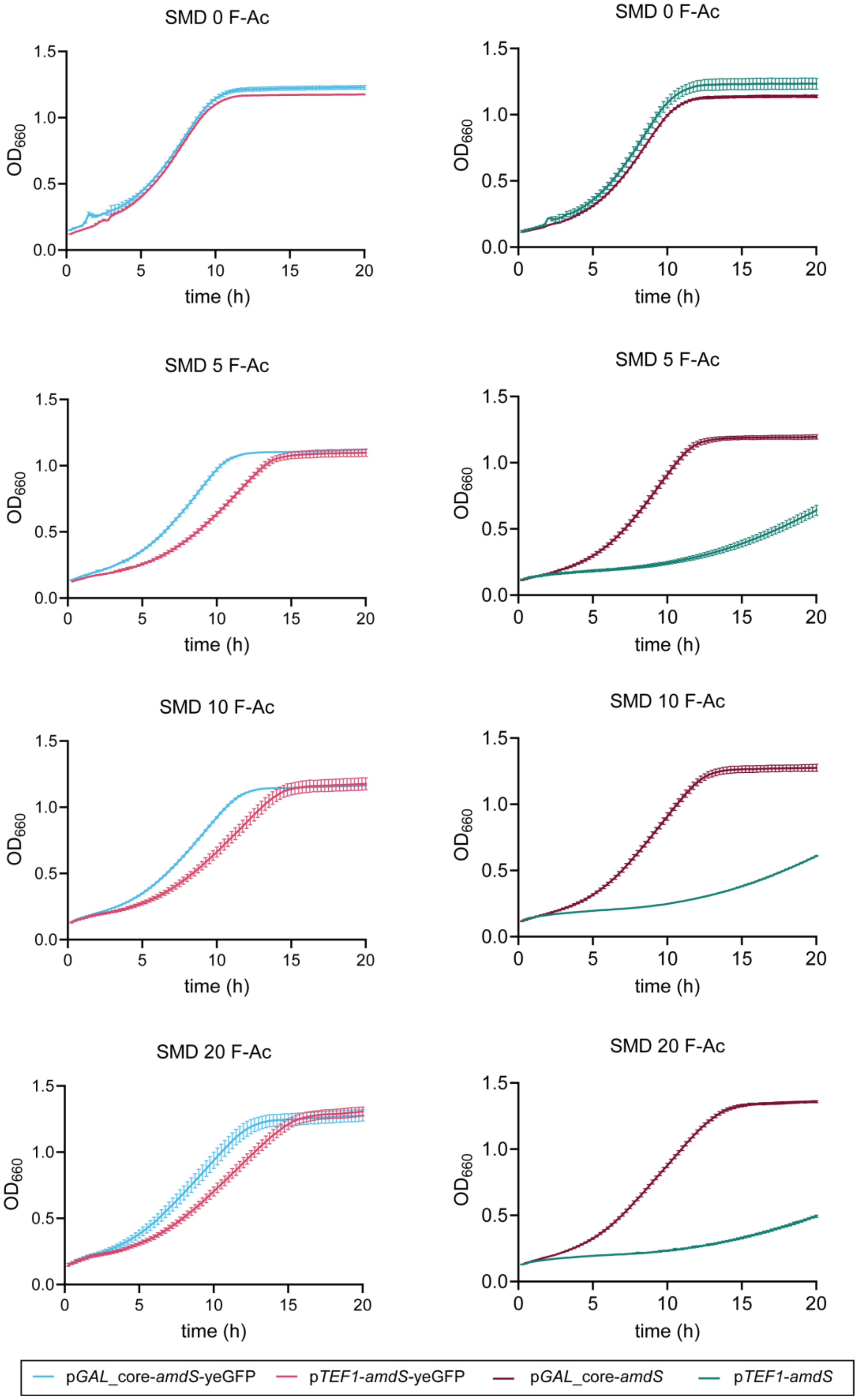

Figure S5: Testing different concentrations of fluoro-acetamide for counter-selection medium. Growth curves of aerobic duplicate batch cultures of *S. cerevisiae* strains expressing *amdS*-yeGFP from a minimal *GAL*-core promoter equipped with 5x*luxO* (blue) (ACA007; *pGAL1_core-5xluxO-amdS-yeGFP*) or

from the strong p*TEF1* (pink) (ACA013; *pTEF1-amdS-yeGFP*) (left panels) and strains expressing *amdS* from a minimal *GAL*-core promoter equipped with 5xluxO (red) (ACA004; *pGAL1_core-5xluxO-amdS*) or from the strong p*TEF1* (green) (ACA002; *pTEF1-amdS*) (right panels) on counter-selection medium; ie. SMD supplemented with different concentrations of fluoro-acetamide (F-Ac). Growth measurements were obtained by a plate reader.

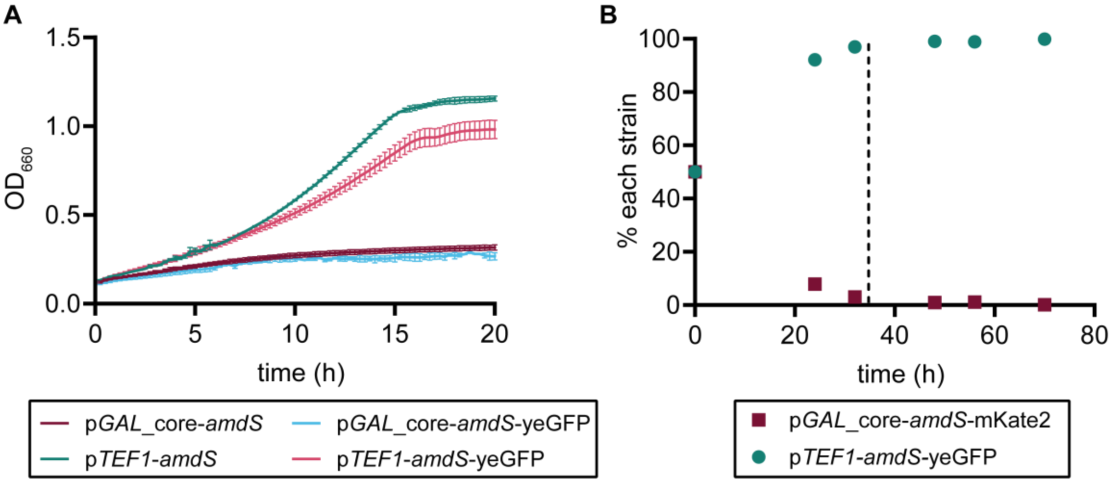

Figure S6: Testing *S. cerevisiae* strains with different expression levels of *amdS* and fusion *amdS* in selection medium. (A) Growth curves of duplicate batch cultures of *S. cerevisiae* strains expressing *amdS*-yeGFP from a minimal *GAL*-core promoter equipped with 5xluxO (blue) (ACA007; *pGAL1_core-5xluxO-amdS-yeGFP*) or from the strong p*TEF1* (pink) (ACA013; *pTEF1-amdS-yeGFP*) and strains expressing *amdS* from a minimal *GAL*-core promoter equipped with 5x*luxO* (red) (ACA004; *pGAL1_core-5xluxO-amdS*) or from the strong p*TEF1* (green) (ACA002; *pTEF1-amdS*) on selection medium; ie. SMD without nitrogen source supplemented with acetamide. Growth measurements were obtained by a plate reader. (B) Strains ACA013 (p*TEF1-amdS*-yeGFP) and ACA008 (p*GAL*_core-*amdS*-mCherry) were co-cultured with a starting ratio of 1:1 on selection medium. Abundance of each strain was determined by flow cytometric analysis. The dotted line indicates 1:100 dilution in fresh counter selection medium. Experiment was performed with biological duplicates, mean and standard deviation of the mean are shown.

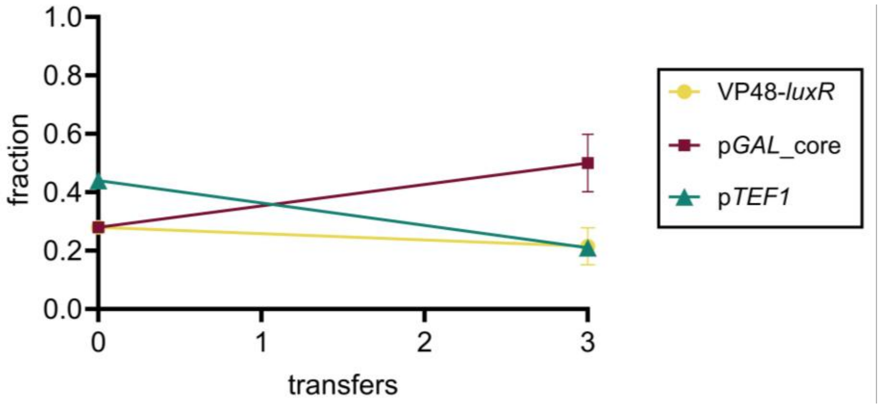

Figure S7: Determining abundance of *S. cerevisiae* strains with different expression levels of *amdS* after 3 consecutive transfers on non-selective medium. Mixture of *S. cerevisiae* strains expressing *amdS* from a minimal *GAL*-core promoter equipped with 5x*luxO* (red) (ACA004), from the strong p*TEF1* (green) (ACA002) and from a minimal *GAL*-core promoter equipped with 5x*luxO* controlled by VP48-LuxR (yellow) (ACA019) were grown on non-selective (ie. SMD) medium for 3 consecutive transfers. The abundance of each strain was determined using colony PCR of 24-32 single colonies at the start of the experiment and after 3 transfers. Experiment was performed in duplicate, and mean values and standard deviation of the mean are shown.

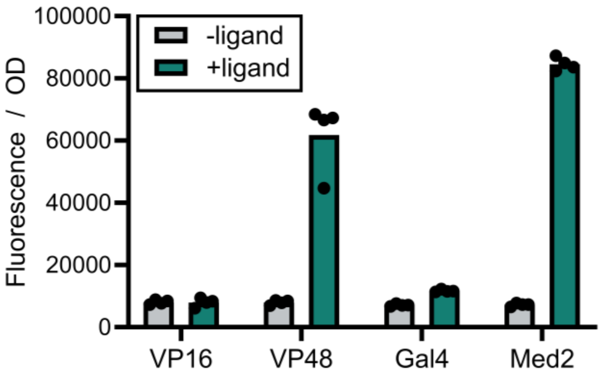

Figure S8: Testing different activation domains N-terminally fused to LuxR. *S. cerevisiae* strains AAA105 (p*GAL1*_5x*luxO*-yeGFP + VP16-*luxR*), AAA036 (p*GAL1*_5xluxO-yeGFP + VP48-*luxR*), AAA101 (p*GAL1*_5x*luxO*-yeGFP + *GAL4*_AD-*luxR*) and AAA103 (p*GAL1*_5x*luxO*-yeGFP + *MED2-luxR*) were grown on SC-complete medium with and without ligand (5 µM 3-oxo-C6-HSL). Fluorescence was measured using a plate reader after 6 h of growth, and the average of 3 consecutive measurements was normalized to OD_{660} for each of 4 biological replicates.

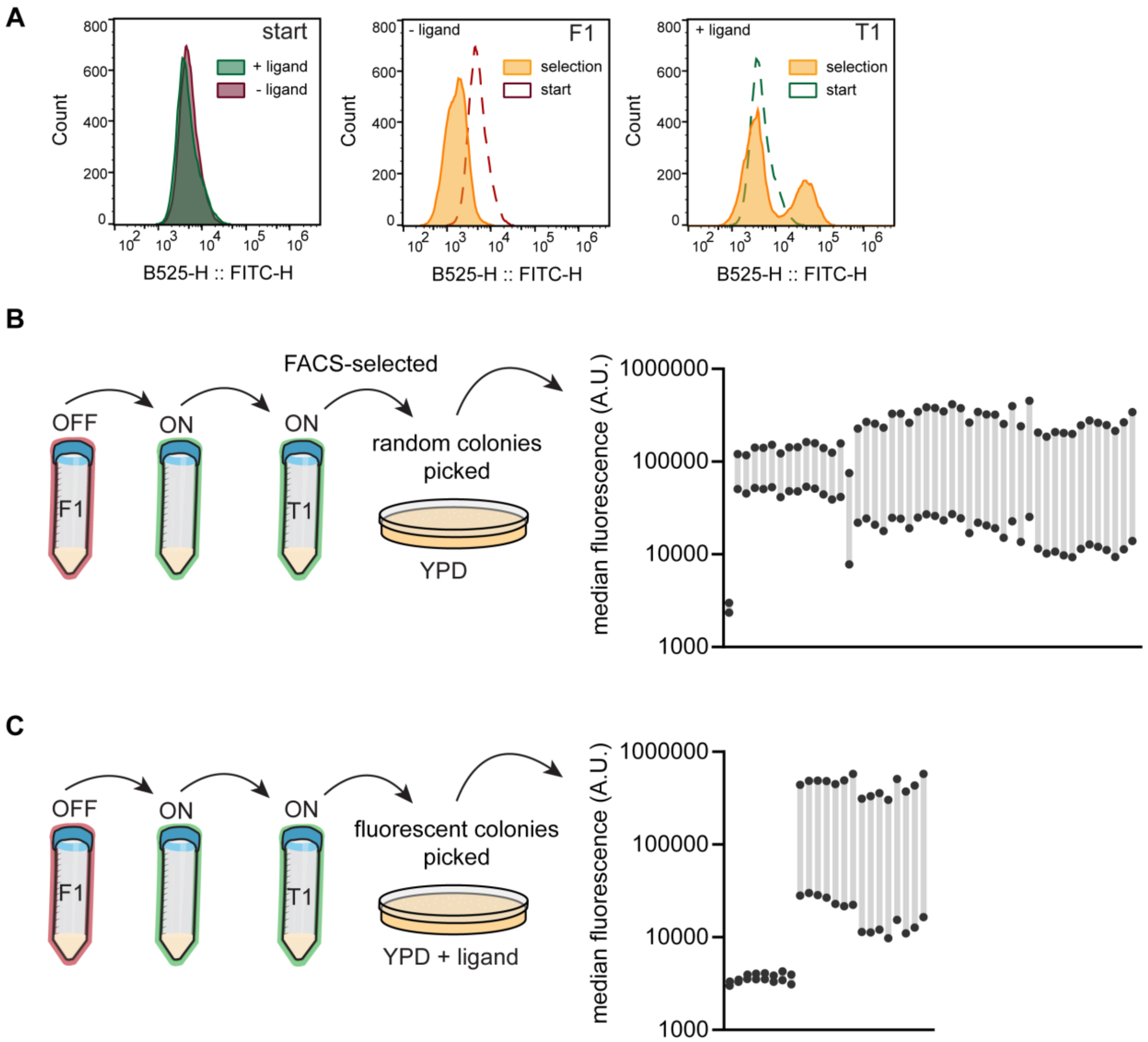

Figure S9: Analysis of single mutants enriched using counter-selection medium and selection medium, followed by FACS-sorting or manual selection using plates. (A) A library of *GAL4*_AD-*luxR*-variants was integrated into a yeast strain containing *amdS* and yeGFP under control of p*GAL*_core_5x*luxO*. The library was grown for 3 days on transformation selection media (SC -HIS +NAT) to select transformants for the starting population. The population was then transferred to counter-selection media (OFF; SMD + F-Ac) and grown for 1 day (population F1). Subsequently, cells were transferred twice on selection media (ON; SMD -N + acetamide), containing 50 nM C8-HSL to obtain population T1. Population fluorescence of each culture was analysed by flow cytometry after 6 hours of growth on SC with and without 50 nM C8-HSL. (B) The 7% most fluorescent cells from population T1 were sorted using FACS, plated for single colonies and 48 colonies were randomly picked. Each colony was analysed by flow cytometry after growing with and without ligand, to determine the dynamic range of GFP expression. (C) Dilutions of culture T1 were plated on YPD supplemented with 50 nM C8-HSL and grown for 24 h. 24 single colonies were selected from the plate, based on visible fluorescence, and analysed by flow cytometry after growing with and without ligand, to determine the dynamic range of yeGFP expression. A.U.: arbitrary units.

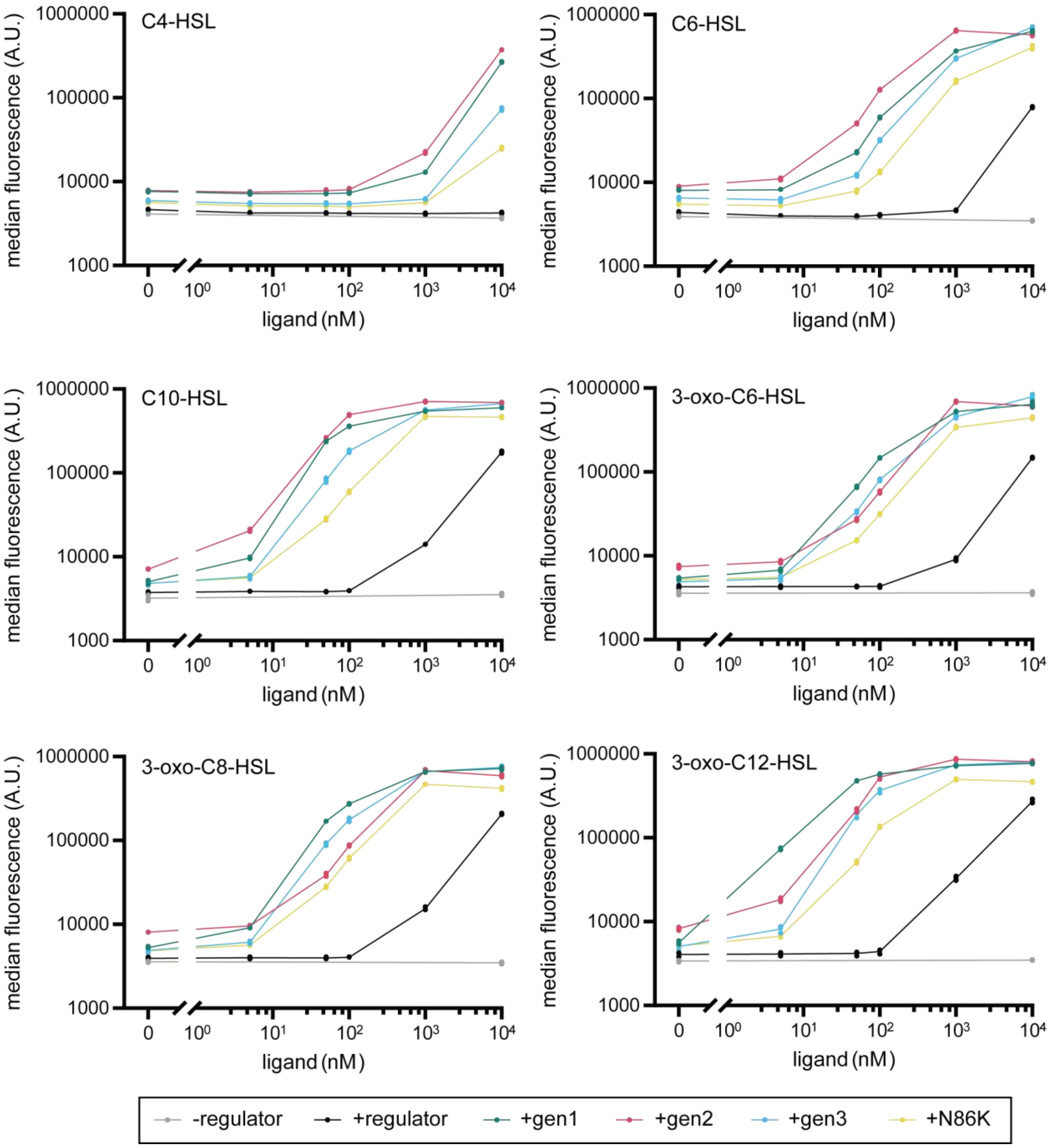

Figure S10: Characterization of Gal4_AD-LuxR-mutants. Dose-response curves of yeGFP fluorescence levels 6 hours following supplementation of 0-10,000 nM of C4-HSL, C6-HSL, C10-HSL, 3-oxo-C6-HSL, 3-oxo-C8-HSL and 3-oxo-C12-HSL, respectively. Tested for *S. cerevisiae* strains AAA036 (- regulator), AAA101 (+ regulator), AAA151 (+ gen1), AAA150 (+ gen2), AAA149 (+ gen3), AAA107 (+ N86K), performed with three biological replicates. A.U.: arbitrary units.

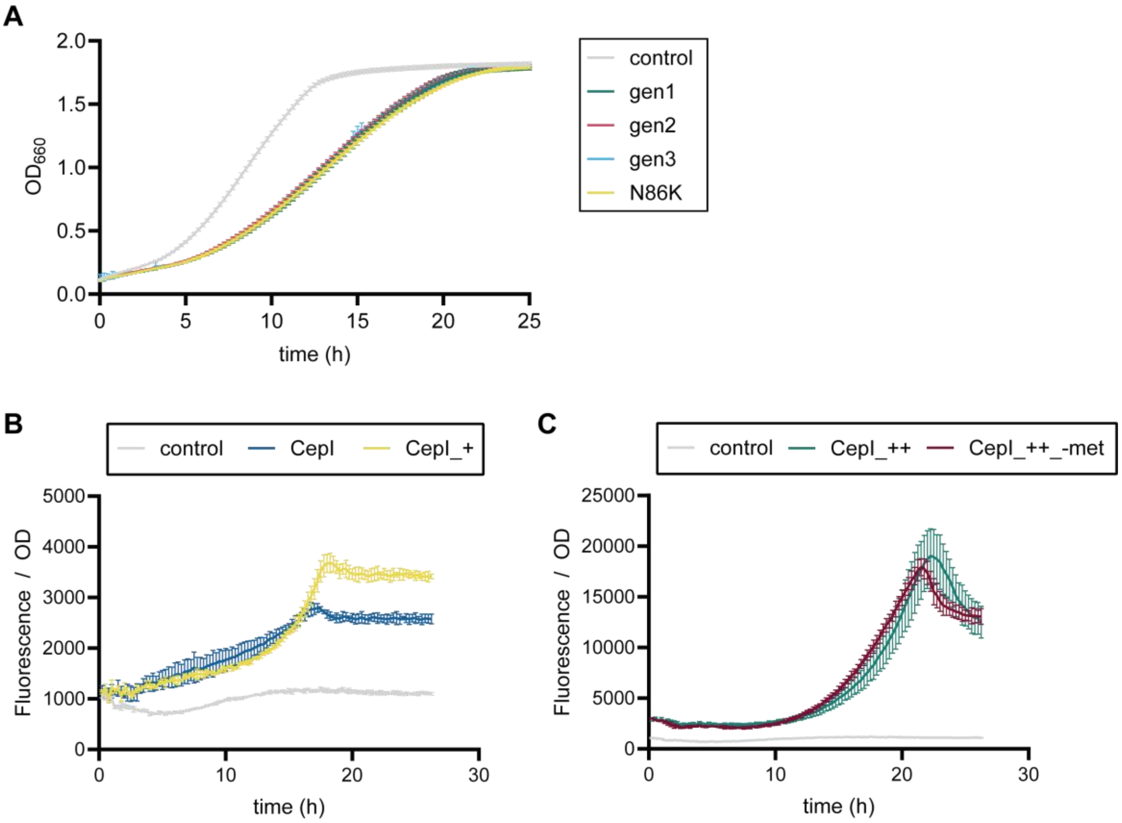

Figure S11: QS-controlled expression of yeGFP in different yeast backgrounds and on different medium compositions. (A) OD_{660} measurements corresponding to Figure 4D of the main manuscript. Fluorescence and OD_{660} were monitored in fluorescence plate reader of *S. cerevisiae* strains AAA148 (CepI (++) + N86K), AAA149 (CepI (++) + gen3), AAA150 (CepI (++) + gen2), AAA151 (CepI (++) + gen1) and control strain AAA189 (CepI (++) – regulator). Cultures were grown on fed-batch medium (EnPump). (B) Fluorescence normalized to OD_{660} on fed-batch medium supplemented with 1 gL^{-1} of methionine of different *S. cerevisiae* strains in fluorescence plate reader. Where indicated, strains express a *Bc.cepI* expression cassette alone (AAA168; CepI) or with an overexpression cassette for *Sc.SAM2* and *Sc.MET6* driven by p*ACT1* and p*PGI1* (AAA169; CepI (+)). All strains contain yeGFP cassette regulated by *GAL4*_AD-l*uxR*_gen1 except for the control strain which expresses a *Bc.cepI* expression cassette, contains an overexpression cassette for *Sc.SAM2* and *Sc.MET6* driven by p*TEF1* and p*PGK1* and contains yeGFP cassette but lacks any regulator (AAA189; control). (C) Strain AAA151 was grown both with and without methionine, as indicated by (CepI_++_-met). Experiments were performed with 3-4 biological replicates.

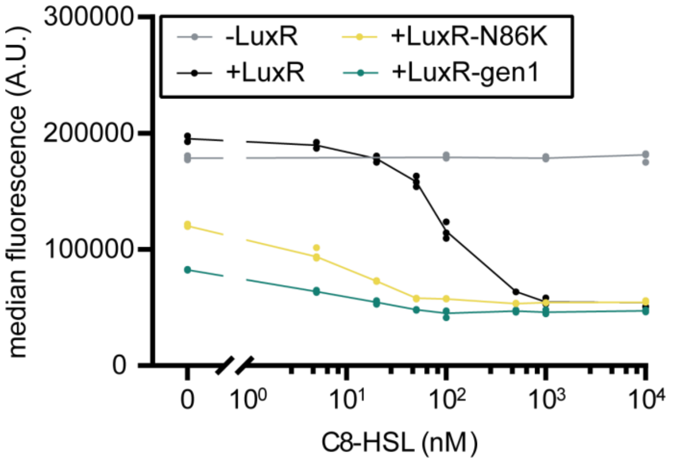

Figure S12: Dose-response curves of *luxR* driving the expression of yeGFP behind p*TEF1* with 2x *luxO* sequence inserted downstream of the TATA-box (p*TEF1_2xluxO*). Dose-response curves of yeGFP fluorescence following a 24 h pre-incubation with 5-10,000 nM C8-HSL prior to transfer to fresh medium with supplementation for 6 h with 5-10,000 nM C8-HSL. Tested for *S. cerevisiae* strains AAA164 (-LuxR), AAA165 (+LuxR), AAA166 (+LuxR-N86K) and AAA172 (+LuxR-gen1) performed with 2-3 biological replicates. A.U.: arbitrary units.

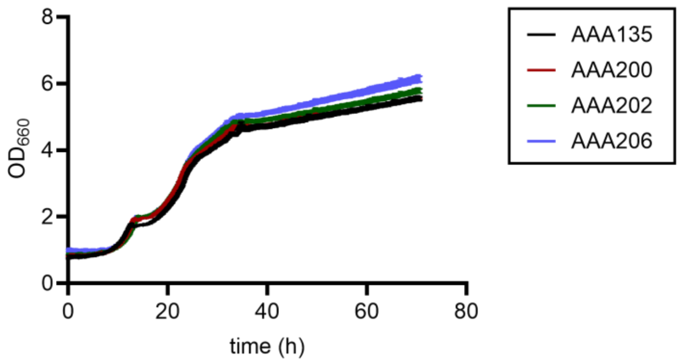

Figure S13: Growth profiles obtained in Biolector plates in glucose fedbatch mode of engineered yeast strains. OD_{660} measurements of aerobic fed-batch cultivation performed in flowerplates analysed by biolector (n=4-5) with strains AAA135 (PKS3), AAA206 (PKS3, CepI (++)), AAA200 (PKS3, LuxR-gen1) and AAA202 (PKS3, LuxR-gen1, CepI (++)).

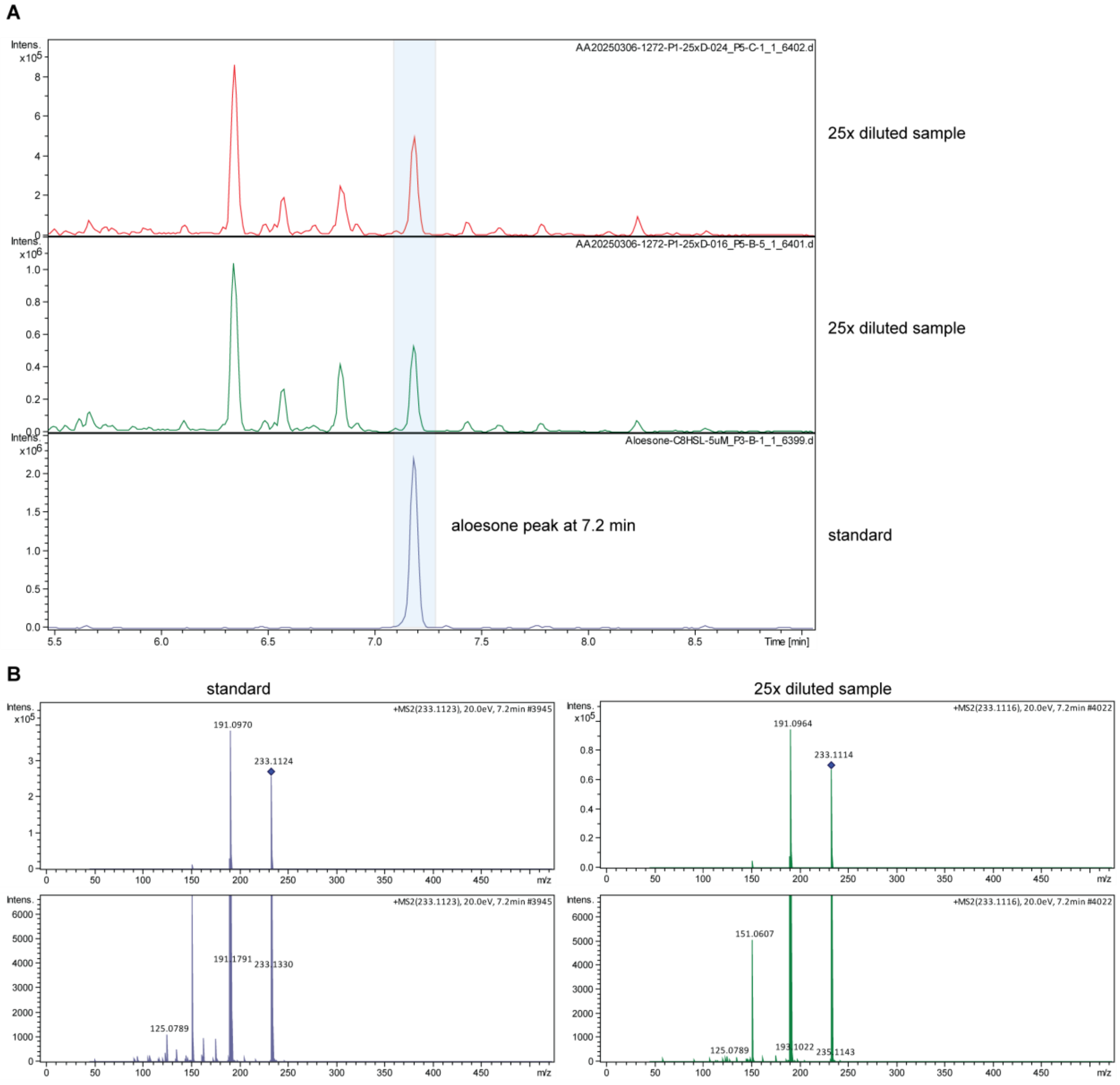

Figure S14: aloesone peak at 7.2 min and MS2 fragmentation pattern. (A) LC-MS chromatogram of 25 times diluted representative samples from end-point determination of aloesone production in aerobic fed-batch cultivation performed in flowerplates analysed by biolector, compared to aloesone standard. Aloesone eluted at a retention time of 7.2 min. (B) MS/MS fragmentation spectra of aloesone (m/z 233.1) acquired at 7.2 min using electrospray ionization in positive mode on a QTOF mass spectrometer at 20 eV collision energy. Major fragment ions include m/z 191.1 (loss of C_2H_4O), 151.1 (loss of C_4H_6O) and 125.1 (further fragmentation), supporting identification of aloesone. Spectra shown from both 25 times diluted experimental sample and aloesone standard.

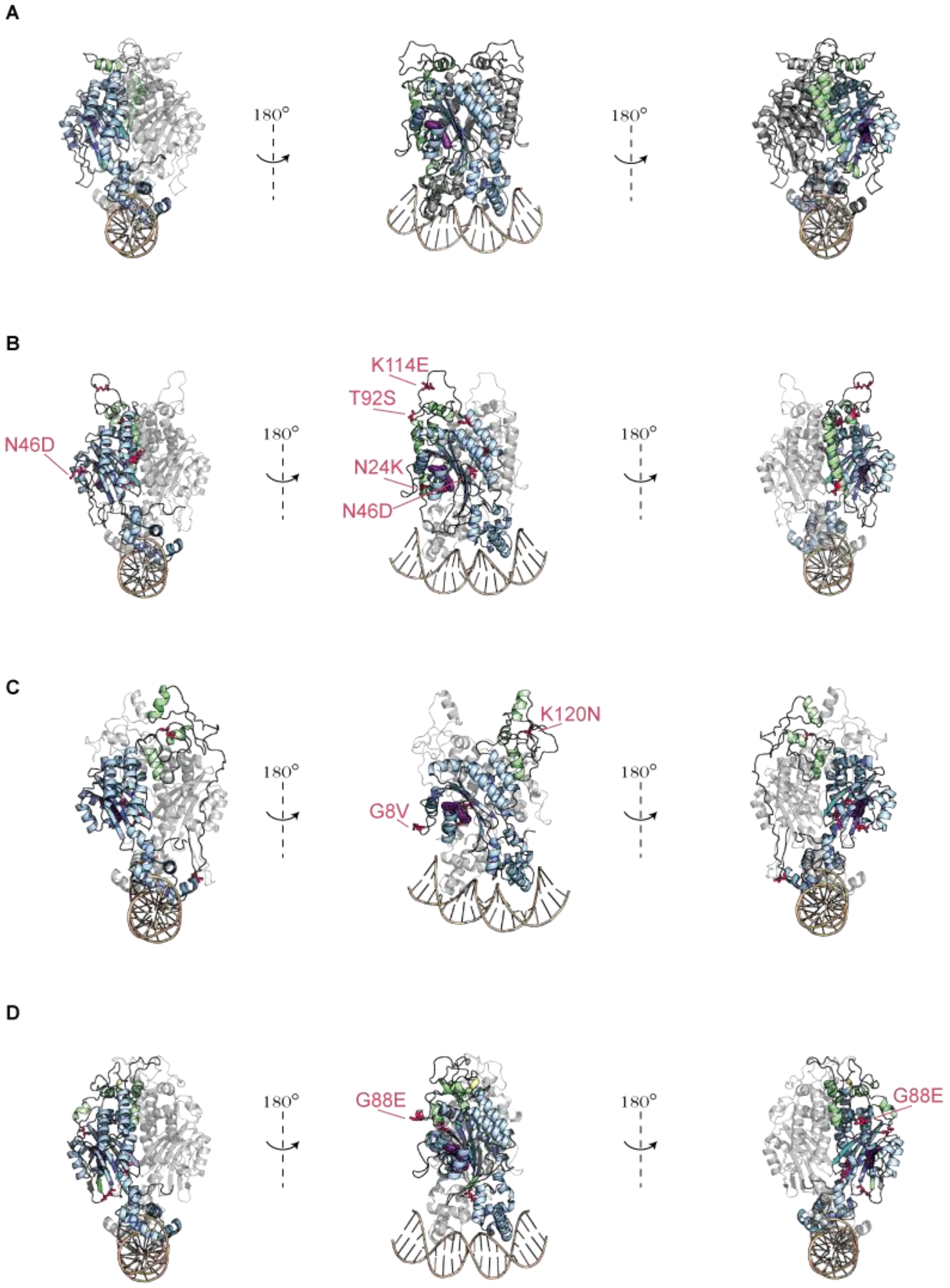

Figure S15: Predicted protein structures of Gal4_AD-NLS-LuxR and mutants. Alphafold3 [1] predicted protein structure of (A) Gal4_AD-NLS-LuxR (B) and structural mapping of mutations found in gen1, (C) gen2 and (D) gen 3 in Gal4_AD-NLS-LuxR. Mutated residues are highlighted in pink and specific mutations in Gal4_AD and NLS are indicated. Gal4_AD is shown in lightblue and LuxR in green, dimer is shown in grey.

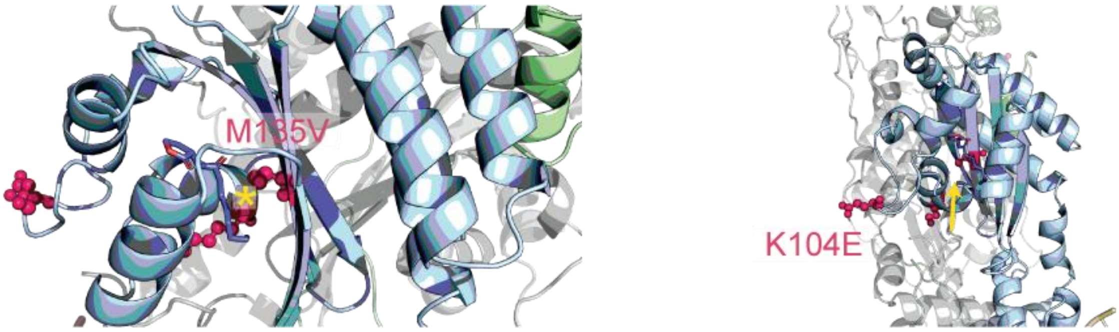

Figure S16: Analysis of protein ligand interaction of LuxR_gen2 with C8-HSL. LuxR_gen2-mutations are shown in pink, asterisk (*) denotes the carbon position that would bear a 3-oxo-group in 3-oxo-C8-HSL and arrow indicates the tunnel entrance of the ligand.

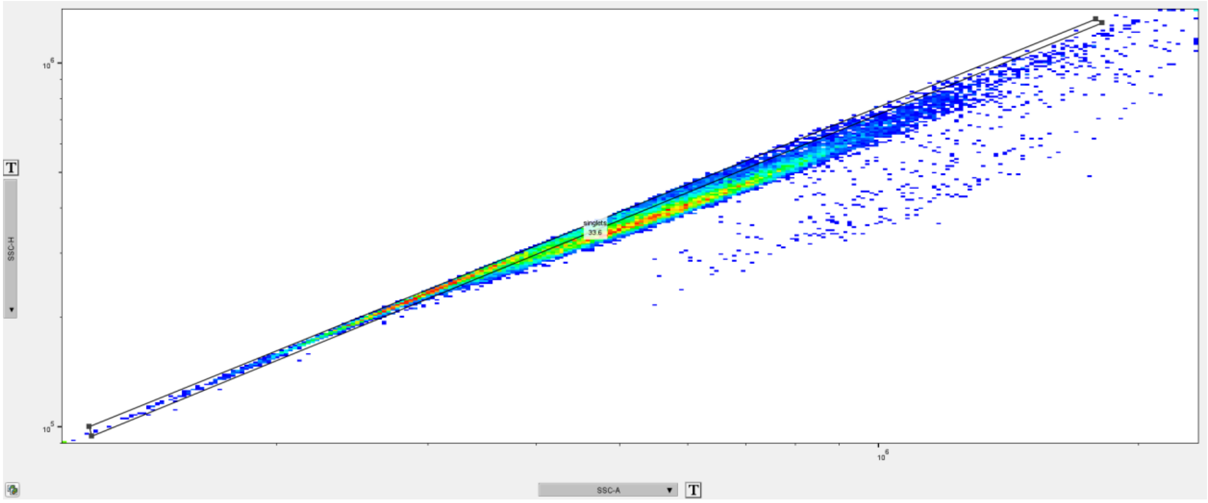

Figure S17: Example of gating strategy of flow cytometry data. FSC-A was plotted against FSC-H to gate for singlets events.

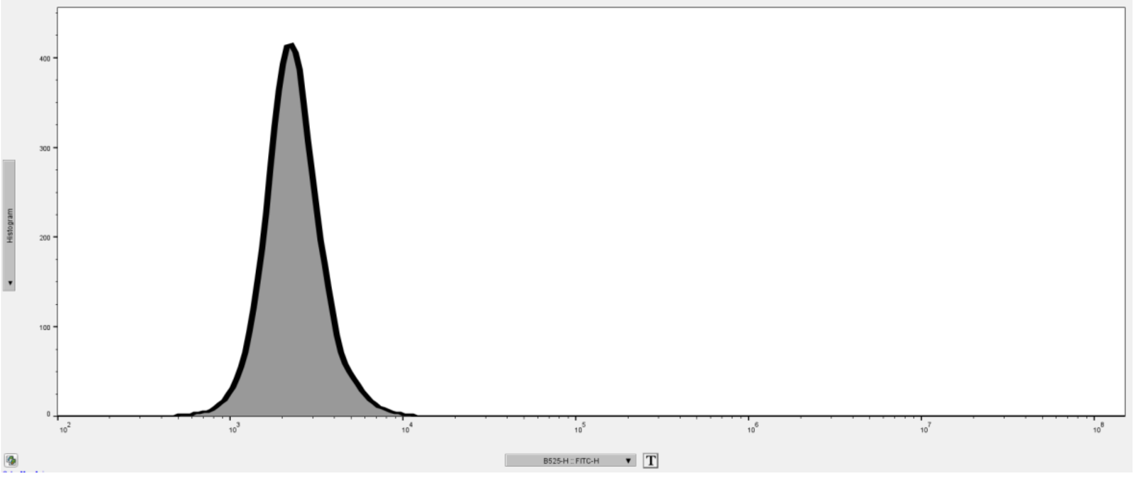

Figure S18: Example of fluorescent events distribution after gating of flow cytometry data. Histogram of fluorescent events.

Table S1: overview of primers used in this study to create linear repair fragments for genomic integrations

name	target	sequence
AA73	p*PGK1*_ep_fw	GATCATCAAGGAAGTAATTATCTACTTTTTACAACAAATATAAAACAACCTGCACTAAAACAATG
AA74	p*PGK1*_fw	GGTCTTTTCTAATTCGTAGTTTTTCAAGTTCTTAGATGCTTTCTTTTTCTCTTTTTTACAGATCATCAAGGAAGTAATTATCTACTTTTT
AA75	p*PGK1*_rv	CATTGTTTTAGTGCAGGTTGTTTTATATTTG
AA76	X-4_UP_fw	CCCAAAGCTAAGAGTCCCATT
AA77	t*ADH1*_ep_rv	AATCATAAATCATAAGAAATTCGCTTATTTAGAAGTGTCAACAACGTATCTACATCTGTCCT
AA78	t*ADH1*_rv	CTAAGAGTCACTTTAAAATTTGTATACACTTATTTTTTTTATAACTTATTTAATAATAAAAATCATAAATCATAAGAAATTCGCTTATTT
AA79	t*ADH1*_fw	AGGACAGATGTAGATACGTTGTTG
AA80	X-4_DN_rv	CTGGTGAGGATTTACGGTATGATC
AA153	*luxR*_N86K_rv	GAATGATTAGACTTGGAGTAATCAACGATTGGGTCG
AA154	*luxR*_N86K_fw	CGTTGATTACTCCAAGTCTAATCATTCTCCCATCAACTGG
AA164	p*FAS1*_p*TEF1*_fw	GCCCGTGCACCCACGGCTCCTCGGAGGCCGGGTTATAGCAGCGTCTGCTCCGCATCACGATACACGAGGTGCAGGCAGCACACACCATAGCTTCAAAATG
AA167	*FAS1*_rv	TGGTACCAGAAGCACGTGCTCTAAAGAACCGTGAGATAGGGTTAATGGTCTTGTGGAGTAAGCGTCCATTGTTTTAGTGCAGGTTTGTAATTAAAACTTA

Table S2: overview of primers used in this study to create plasmids using USER-cloning

name	target	sequence
AA1	*cepI*_fw	ACCTGCACUAAAACAATGCAAACTTTTGTTCATGAAGAAGGTAG
AA2	*cepI*_rv	ATCTGTCCUTGAAGCAGCAATAGCTTGTCTAGC
AA9	*lasI*_fw	ACCTGCACUAAAACAATGATTGTTCAGATTGGCCGTCG
AA10	*lasI*_rv	ATCTGTCCUTTAGGAAACGGCCAGGCG
AA13	*luxI*_fw	ACCTGCACUAAAACAATGACTATAATGATAAAAAAATCGGATTTTTTGGC
AA14	*luxI*_rv	ATCTGTCCUTTAATTTAAGACTGCTTTTTTAAACTGTTCATTAATAG
AA15	*luxR*_fw	ACCTGCACUAAAACAATGGAAAACATCAACGCTGATGACAC
AA16	*luxR*_rv	ATCTGTCCUTTAATTCTTAAAGTATGGACAATCAATAGCACC
AA17	VP48_fw	ACCTGCACUAAAACAATGCCAGCCGATGCTTTG
AA34	p*TEF1*_fw	AGTGCGAUGCACACACCATAGCTTCAAAATG
AA35	p*TEF1*_rv	AGTGCAGGUTTGTAATTAAAACTTAGATTAGATTGCTATGCTTTC
AA36	p*GAL*_core_fw	AGTGCGAUAAAACTGTATTATAAGTAAACTGAAAAAGCGTG
AA37	p*GAL*_core_rv	AGTGCAGGUGTCGACGCTAGCTATAGTTTTTTCTCC
AA81	*amdS*_fw	ACCTGCACUAAAACAATGCCACAATCTTGGGAAGAATTGG
AA82	G43S_*amdS*_rv	ATGCTAGCUGATCCGCCCCCACCAGACCCACCTCCGCCAGAGCCTCCACCCCCTTATGGAGTAACAACGTTACCCAAC
AA83	G43S_mKate2_fw	AGCTAGCAUGGTTTCTGAACTCATCAAGGAAAAC
AA85	G43S_yeGFP_fw	AGCTAGCAUGTCTAAAGGTGAAGAATTATTCACTGG
AA86	mKate2_rv	ATCTGTCCUTTATCTGTGTCCCAACTTAGATGGC
AA93	p*ACT1*_rv	ACCTGCACUTGTTAATTCAGTAAATTTTCGATCTTGGGAAG
AA95	p*PGI1*_rv	ATGACAGAUTTTTAGGCTGGTATCTTGATTCTAAATCG
AA103	esaI_co_fw	ACCTGCACUAAAACAATGTTGGAGTTGTTCGATGTCTCC
AA104	esaI_co_rv	ATCTGTCCUTTAAACTGGTAAAGTCAATGGCCAAG
AA105	*lasI*_co_fw	ACCTGCACUAAAACAATGATCGTCCAAATCGGTAGAAGAG
AA106	*lasI*_co_rv	ATCTGTCCUTTAAGAAACGGCCAATCTTTGTTCAAC
AA107	*luxI*_co_fw	ACCTGCACUAAAACAATGACCATCATGATCAAGAAGTCCG
AA108	*luxI*_co_rv	ATCTGTCCUTTAGTTCAAAACGGCCTTCTTGAATTG
AA121	*amdS*_rv	ATCTGTCCUTTATGGAGTAACAACGTTACCCAAC
AA129	p*ACT1*_fw	ATCGCACUGAAGCGGGTAAGCTGCCAC
AA130	p*PGI1*_fw	AGTGCGAUAGAGAATTTTGCCATCGGACATGCTACCTTACGC
AA131	*SAM2*_fw	AGTGCAGGUAAAACAATGTCCAAGAGCAAAACTTTCTTATTTAC
AA132	*SAM2*_rv	CGTGCGAUTTAAAATTCCAATTTCTTTGGTTTTTCCC
AA135	NLS1_*luxR*_fw	AGCCAGGUCCACCAAAAAAGAAG
AA136	NLS1_*GAL4*_AD_rv	ACCTGGCUCTTTTTTTGGGTTTGGTGGGG
AA137	*GAL4*_AD_fw	ACCTGCACUAAAACAATGGCCAATTTTAATCAAAGTGGGAATATTGC

AA138	NLS2_*luxR*_fw	ACCAGGUCCACCAAAAAAGAAG
AA139	NLS2_*MED2*_rv	ACCTGGUATATTGAAGCCGCTGAGGTCTAATTC
AA140	*MED2*_fw	ACCTGCACUAAAACAATGGTAGTACAAAATAGCCCAGTTTCGTC
AA141	VP16_fw	ACCTGCACUAAAACAATGCCAGCAGACGCATTGGATGAC
AA144	p*TEF1*_fw	ATCGCACUGCACACACCATAGCTTCAAAATG
AA145	p*TEF1*_rv	ACCTGCACUTTGTAATTAAAACTTAGATTAGATTGCTATGCTTTC
AA161	*MET6*_fw	ATCTGTCAUAAAACAATGGTTCAATCTGCTGTCTTAGGG
AA162	*MET6*_rv	CACGCGAUTTAATTCTTGTATTGTTCACGGAAGTACTTG
AA180	gRNA_p*FAS1*_fw	TTGTTGCTATTTAACGCTTTGTTTTAGAGCTAGAAATAGCAAGTTAAAATAAGGC
MAD3	BB_fw	ATCGCAUATCGCACGCATTCCATGCGA
MAD4	BB_rv	ACCGCGTUATCGCGTACCAATTCGCCC
MAD12	yeGFP_fw	ACCTGCACUAAAACAATGTCTAAAGGTGAAGAATTATTC
MAD13	yeGFP_rv	ATCTGTCCUTTATTTGTACAATTCATCC
MAD14	t*CPS1*_fw	AGGACAGAUGCGCAATGATTGAATAGTC
MAD15	t*CPS1*_rv	AACGCGGUGTGGTTTTGATTTGATTTAAGTC
MAD36	p*PGK1*_fw	AGTGCGAUAGACGCGAATTTTTCGAAGAAG
MAD37	p*PGK1*_rv	AGTGCAGGUTGTTTTATATTTGTTGTAAAAAGTAGATAATTAC
MAD38	p*TDH3*_fw	AGTGCGAUCTATTTTCGAGGACCTTGTCACC
MAD39	p*TDH3*_rv	AGTGCAGGUTTTGTTTGTTTATGTGTGTTTATTCG
MAD61	t*CYC1*_rv	AACGCGGUCTTCGAGCGTCCCAAAACCTTCTC
MAD62	t*CYC1*_fw	AGGACAGAUATCCGCTCTAACCGAAAAGG
MAD63	t*ADH1*_fw	AGGACAGAUGTAGATACGTTGTTGACACTTC
MAD64	t*ADH1*_rv	AACGCGGUGAGCGACCTCATGCTATACC
TJOS-20	gRNA_bb_rv	GATCATTTATCTTTCACTGCGGAGAAG

Table S3: **overview of plasmids used in this study**

name	relevant genotype	details plasmid construction	source
pTAJAK-161	Cas9, HIS	-	[2]
pCfB3045	XI-3 gRNA, NAT	-	[3]
pCfB6915	XI-3 gRNA, URA	-	[4]
pCfB3042	X-4 gRNA, NAT	-	[3]
pCfB6912	X-4 gRNA, URA		[4]
pCfB9340	VIII-1 gRNA, NAT	-	[5]
pCfB3050	XII-5 gRNA, NAT	-	[3]
pCfB3020	X-2 gRNA, NAT	-	[3]
pCfB6910	X-2 gRNA, URA	-	[4]
pCfB2904	XI-3 USER overhangs	-	[3]
pCfB3035	X-4 USER overhangs	-	[3]
pCfB2909	XII-5 USER overhangs	-	[3]
pCfB2899	X-2 USER overhangs	-	[3]
pCfB9359	VIII-1 USER overhangs	-	[5]
pAvA001	*cepI* (codon-optimized)	Synthesized by IDT	This study
LuxTA2-4F	VP48-NLS-*luxR*	Ordered from addgene	[6]
pLuxO5	p*GAL*_core-5xluxO	Ordered from addgene	[6]
pLas81-LuxI (#73445)	*luxI*	Ordered from addgene	[7]
pLux76-LasI (#73444)	*lasI*	Ordered from addgene	[7]
pAC-EsaR-EsaI (#47660)	esaI	Ordered from addgene	[8]
pAvA014	XI-3-p*TEF*_luxO*_105-yeGFP-t*CPS1*	USER-cloning	This study
pAvA026	XI-3-p*GAL*1-5xLuxO-yeGFP-t*CPS1*	USER-cloning	This study
pAvA027	X-4-p*PGK1*-*luxR*-t*ADH1*	USER-cloning	This study
pAvA028	X-4-p*PGK1*-VP48-NLS-*luxR*-t*ADH1*	USER-cloning	This study
pAvA030	X-2-p*TDH3*-*luxI*-t*CYC1*	USER-cloning	This study
pAvA031	X-2-p*TDH3*-*lasI*-t*CYC1*	USER-cloning	This study
pAvA045	X-2-p*TDH3*-esaI-t*CYC1*	USER-cloning	This study
pAvA055	XI-3-p*TEF*-*amdS*-yeGFP-t*CPS1*	USER-cloning	This study

pAvA061	XI-3-p*GAL*_core-5xluxO-*amdS*-yeGFP-t*CPS1*	USER-cloning	This study
pAvA063	XI-3-p*GAL*_core-5xluxO-*amdS*-mKate2-t*CPS1*	USER-cloning	This study
pAvA068	X-2-p*TDH3*-*cepI* (co)- tCYC	USER-cloning	This study
pAvA071	esaI (codon-optimized)	Synthesized by Thermo Scientific	This study
pAvA073	*lasI* (codon-optimized)	Synthesized by Thermo Scientific	This study
pAvA074	*luxI* (codon-optimized)	Synthesized by Thermo Scientific	This study
pAvA078	X-2-p*TDH3*-*luxI* (co)-t*CYC1*	USER-cloning	This study
pAvA079	X-2-p*TDH3*-esaI (co)-t*CYC1*	USER-cloning	This study
pAvA080	X-2-p*TDH3*-*lasI* (co)-t*CYC1*	USER-cloning	This study
pAvA085	XI-3-p*TEF1*-*amdS*-t*CPS1*	USER-cloning	This study
pAvA087	XI-3-p*GAL*_core-5xluxO-*amdS*-t*CPS1*	USER-cloning	This study
pAvA096	PKS3 (codon-optimized)	Synthesized by Thermo Scientific	This study
pAvA098	p*TEF1*_luxO_105	Synthesized by Thermo Scientific	This study
pAvA100	XII-5-p*PGK1*-Aa.PKS3 (co)-tCYC	USER-cloning	This study
pAvA102	XI-3-p*TEF*_luxO_105-yeGFP-t*CPS1*	USER-cloning	This study
pAvA103	X-4-p*PGK1*-*GAL4*_AD-NLS-*luxR*-t*ADH1*	USER-cloning	This study
pAvA105	X-4-p*PGK1*-*MED2*-NLS-*luxR*-t*ADH1*	USER-cloning	This study
pAvA107	X-4-p*PGK1*-VP16-NLS-*luxR*-t*ADH1*	USER-cloning	This study
pAvA109	XI-3-p*TEF1*_luxO_105-*FAS1*-t*CPS1*	USER-cloning	
pAvA117	XII-5-p*GAL*_core-5xluxO-yeGFP-t*CPS1*	USER-cloning	This study
pAvA118	XII-5-t*ADH1*-*SAM2*-p*TEF1*--p*PGK1*-*MET6*-t*CYC1*	USER-cloning	This study
pAvA119	XII-5-t*ADH1*-*SAM2*-p*ACT1*--p*PGI1*-*MET6*-t*CYC1*	USER-cloning	This study
pAvA125	gRNA_p*FAS1*	USER-cloning	This study
pAvA134	XI-3-p*TEF1*_2xluxO_98_105-eGFP-t*CPS1*	USER-cloning	
pAvA139	X-4-p*PGK1*-[*GAL4*_AD-NLS-l*uxR*]_gen1-t*ADH1*	USER-cloning	This study
pAvA141	X-4-p*PGK1*-[*GAL4*_AD-NLS-l*uxR*]_gen2-t*ADH1*	USER-cloning	This study
pAvA143	X-4-p*PGK1*-[*GAL4*_AD-NLS-l*uxR*]_gen3-t*ADH1*	USER-cloning	This study
pAvA155	VIII-1-t*ADH1*-*SAM2*-p*TEF1*--p*PGK1*-*MET6*-t*CYC1*	USER-cloning	This study

Table S4: overview of yeast strains used in this study and details on strain construction

**name**	**relevant genotype**	**details strain construction**	**source**
**CEN.PK2-1C**	*MAT*-a *ura3 his3 leu2 trp1*	-	[9]
**CEN.PK110-10C**	*MAT*-α *URA3 his3 LEU2 TRP1*	-	[9]
**AAA001**	*p-Cas9*	CEN.PK2-1C: pTAJAK-161	This study
**AAA005**	*MAT*-a *ura3 his3 leu2 trp1 XI-3-pTEF_luxO*-yeGFP-tCPS1 p-Cas9*	AAA001: pCfB6915 + NotI digested pAvA014	This study
**AAA010**	*MAT*-a *ura3 his3 leu2 trp1 XI-3-pGAL1-5xluxO-yeGFP-tCPS1 p-Cas9*	AAA001: pCfB6915 + NotI digested pAvA026	This study
**AAA014**	*MAT*-a *ura3 his3 leu2 trp1 XI-3-pTEF_luxO*-yeGFP-tCPS1*	AAA005: plasmids removed	This study
**AAA019**	*MAT*-a *ura3 his3 leu2 trp1 XI-3-pGAL_core-5xluxO-yeGFP-tCPS1*	AAA010: plasmids removed	This study
**AAA035**	*MAT*-a *ura3 his3 leu2 trp1 XI-3-pGAL1_core-5xluxO-yeGFP-tCPS1 X-4-pPGK1-luxR-tADH1*	AAA010: pCfB3042 + NotI digested pAvA027	This study

**AAA036**	*MAT*-a *ura3 his3 leu2 trp1 XI-3-pGAL1_core-5xluxO-yeGFP-tCPS1 X-4-pPGK1-VP48-NLS-luxR-tADH1*	AAA010: pCfB3042 + NotI digested pAvA028	This study
**AAA062**	*MAT*-a *ura3 his3 leu2 trp1 X-2-pTDH3-cepI (co)-tCYC p-Cas9*	AAA001: pCfB3020 + NotI digested pAvA068	This study
**AAA063**	*MAT*-a *ura3 his3 leu2 trp1 X-2-pTDH3-cepI (co)-tCYC*	AAA062: plasmids removed	This study
**AAA070**	*MAT*-a *ura3 his3 leu2 trp1 XI-3-pTEF_luxO*-yeGFP-tCPS1 X-4-pPGK1-luxR-tADH1*	AAA005: pCfB3042 + NotI digested pAvA027	This study
**AAA071**	*MAT*-a *ura3 his3 leu2 trp1 XI-3-pTEF_luxO*-yeGFP-tCPS1 X-4-pPGK1-VP48-NLS-luxR-tADH1*	AAA005: pCfB3042 + NotI digested pAvA028	This study
**AAA093**	*XII-5-pPGK1-AaPKS3_co-tCYC, pCas9*	AAA001: pCfB3050 + NotI digested pAvA100	This study
**AAA095**	*MAT*-a *ura3 his3 leu2 trp1 XI-3-pTEF_luxO-yeGFP-tCPS1 p-Cas9*	AAA001: pCfB6915 + NotI digested pAvA102	This study
**AAA096**	*MAT*-a *ura3 his3 leu2 trp1 XI-3-pTEF_luxO-yeGFP-tCPS1*	AAA095: plasmids removed	This study
**AAA101**	*MAT*-a *ura3 his3 leu2 trp1 XI-3-pGAL1_core-5xLuxO-yeGFP-tCPS1 X-4-pPGK1-GAL4_AD-NLS-LuxR-tADH1*	AAA010: pCfB3042 + NotI digested pAvA103	This study
**AAA103**	*MAT*-a *ura3 his3 leu2 trp1 XI-3-pGAL1_core-5xluxO-yeGFP-tCPS1 X-4-pPGK1-MED2-NLS-LuxR-tADH1*	AAA010: pCfB3042 + NotI digested pAvA105	This study
**AAA105**	*MAT*-a *ura3 his3 leu2 trp1 XI-3-pGAL1_core-5xluxO-yeGFP-tCPS1 X-4-pPGK1-VP16-NLS-LuxR-tADH1*	AAA010: pCfB3042 + NotI digested pAvA107	This study
**AAA107**	*MAT*-a *ura3 his3 leu2 trp1 XI-3-pGAL1_core-5xluxO-yeGFP-tCPS1 X-4-pPGK1-GAL4_AD-NLS-LuxR_N86K-tADH1*	AAA010: pCfB3042 + X-4-p*PGK1-GAL4*_AD-NLS-*luxR*_N86K + *luxR*_N86K-t*ADH1*	This study
**AAA110**	*MAT*-a *ura3 his3 leu2 trp1 X-2-pTDH3-cepI (co)-tCYC XII-5-tADH1-SAM2-pTEF1--pPGK1-MET6-tCYC1 p-Cas9*	AAA062: pCfb3050 + NotI digested pAvA118	This study
**AAA111**	*MAT*-a *ura3 his3 leu2 trp1 X-2-pTDH3-cepI (co)-tCYC XII-5-tADH1-SAM2-pTEF1--pPGK1-MET6-tCYC1 p-Cas9*	AAA110: plasmids removed	This study
**AAA112**	*MAT*-a *ura3 his3 leu2 trp1 X-2-pTDH3-cepI (co)-tCYC XII-5-tADH1-SAM2-pACT1--pPGI1-MET6-tCYC1 p-Cas9*	AAA062: pCfb3050 + NotI digested pAvA119	This study
**AAA113**	*MAT*-a *ura3 his3 leu2 trp1 X-2-pTDH3-cepI (co)-tCYC XII-5-tADH1-SAM2-pACT1--pPGI1-MET6-tCYC1*	AAA112: plasmids removed	This study
**AAA134**	*MAT*-a *ura3 his3 leu2 trp1 XII-5-pPGK1-AaPKS3_co-tCYC pFAS1::pTEF1_luxO p-Cas9*	AA093: pAvA125 + p*TEF1*_luxo_105	This study
**AAA135**	*MAT*-a *ura3 his3 leu2 trp1 XII-5-pPGK1-AaPKS3_co-tCYC pFAS1::pTEF1_luxO*	AAA134: plasmids removed	This study
**AAA148**	*MAT*-a *ura3 his3 leu2 trp1 X-2-pTDH3-CepI-co-tCYC XII-5-tADH1-SAM2-pTEF1--pPGK1-MET6-tCYC1 XI-3-pGAL_core-5xluxO-yeGFP-tCPS1 X-4-pPGK1-GAL4_AD-NLS-luxR_N85K_tADH1*	AAA110: pCfB6915 + NotI digested pAvA026, pCfB3042 + X-4-p*PGK1-GAL4*_AD-NLS-*luxR*_N86K + *luxR*_N86K-t*ADH1*	This study

**AAA149**	*MAT*-a *ura3 his3 leu2 trp1 X-2-pTDH3-CepI-co-tCYC XII-5-tADH1-SAM2-pTEF1--pPGK1-MET6-tCYC1 XI-3-pGAL_core-5xluxO-yeGFP-tCPS1 X-4-pPGK1-GAL4_AD-NLS-luxR_gen3_tADH1*	AAA110: pCfB6915 + NotI digested pAvA026, pCfB3042 + NotI digested pAvA143	This study
**AAA150**	*MAT*-a *ura3 his3 leu2 trp1 X-2-pTDH3-CepI-co-tCYC XII-5-tADH1-SAM2-pTEF1--pPGK1-MET6-tCYC1 XI-3-pGAL_core-5xluxO-yeGFP-tCPS1 X-4-pPGK1-GAL4_AD-NLS-luxR_gen2_tADH1*	AAA110: pCfB6915 + NotI digested pAvA026, pCfB3042 + NotI digested pAvA141	This study
**AAA151**	*MAT*-a *ura3 his3 leu2 trp1 X-2-pTDH3-CepI-co-tCYC XII-5-tADH1-SAM2-pTEF1--pPGK1-MET6-tCYC1 XI-3-pGAL_core-5xluxO-yeGFP-tCPS1 X-4-pPGK1-GAL4_AD-NLS-luxR _gen1_tADH1*	AAA110: pCfB6915 + NotI digested pAvA026, pCfB3042 + NotI digested pAvA139	This study
**AAA156**	*MAT*-a *ura3 his3 leu2 trp1 XI-3-pGAL1_core-5xluxO-yeGFP-tCPS1 X-4-pPGK1-GAL4_AD-NLS-LuxR_gen1-tADH1*	AAA010: pCfB3042 + NotI digested pAvA139	This study
**AAA157**	*MAT*-a *ura3 his3 leu2 trp1 XI-3-pGAL1_core-5xluxO-yeGFP-tCPS1 X-4-pPGK1-GAL4_AD-NLS-LuxR_gen2-tADH1*	AAA010: pCfB3042 + NotI digested pAvA141	This study
**AAA158**	*MAT*-a *ura3 his3 leu2 trp1 XI-3-pGAL1_core-5xluxO-yeGFP-tCPS1 X-4-pPGK1-GAL4_AD-NLS-LuxR_gen3-tADH1*	AAA010: pCfB3042 + NotI digested pAvA143	This study
**AAA163**	*MAT*-a *ura3 his3 leu2 trp1 XI-3-pTEF1_2xluxO_98_105-eGFP-tCPS1, p-Cas9*	AAA001: pCfB3045 + NotI digested pAvA134	This study
**AAA164**	*MAT*-a *ura3 his3 leu2 trp1 XI-3-pTEF1_2xluxO_98_105-eGFP-tCPS1*	AAA163: plasmids removed	This study
**AAA165**	*MAT*-a *ura3 his3 leu2 trp1 XI-3-pTEF1_2xluxO_98_105-eGFP-tCPS1 X-4-pPGK1-luxR-tADH1*	AAA163: pCfB3042 + NotI digested pAvA027	This study
**AAA166**	*MAT*-a *ura3 his3 leu2 trp1 XI-3-pTEF1_2xluxO_98_105-eGFP-tCPS1 X-4-pPGK1-luxR_N86K-tADH1*	AAA163: pCfB3042 + X-4-p*PGK1-luxR*_N86K + *luxR*_N86K-t*ADH1*	This study
**AAA168**	*MAT*-a *ura3 his3 leu2 trp1 X-2-pTDH3-CepI-co-tCYC, XI-3-pGAL_core-5xLuxO-yeGFP-tCPS1 X-4- pPGK1-GAL4_AD-NLS-LuxR _gen1_tADH1*	AAA062: pCfB6915 + NotI digested pAvA026, pCfB3042 + NotI digested pAvA139	This study
**AAA169**	*MAT*-a *ura3 his3 leu2 trp1 X-2-pTDH3-CepI-co-tCYC XII-5-tADH1-SAM2-pACT1--pPGI1-MET6-tCYC1 XI-3-pGAL_core-5xLuxO-yeGFP-tCPS1 X-4-pPGK1-GAL4_AD-NLS-luxR_gen1_tADH1*	AAA112: pCfB6915 + NotI digested pAvA026, pCfB3042 + NotI digested pAvA139	This study
**AAA170**	*MAT*-a *ura3 his3 leu2 trp1 XI-3-pTEF_luxO-eGFP-tCPS1 X-4-pPGK1-luxR-tADH1*	AAA095: pCfB3042 + NotI digested pAvA027	This study

**AAA171**	*MAT*-a *ura3 his3 leu2 trp1 XI-3-pTEF_luxO-eGFP-tCPS1 X-4-pPGK1-luxR_N86K-tADH1*	AAA095: pCfB3042 + X-4-p*PGK1*-*luxR*_N86K + *luxR*_N86K-t*ADH1*	This study
**AAA172**	*MAT*-a *ura3 his3 leu2 trp1 XI-3-pTEF1_2xluxO_98_105-eGFP-tCPS1 X-4-pPGK1-luxR-gen1-tADH1*	AAA163: pCfB9342 + NotI digested pAvA139	This study
**AAA189**	*MAT*-a *ura3 his3 leu2 trp1 X-2-pTDH3-cepI-co-tCYC XII-5-tADH1-SAM2-pTEF1--pPGK1-MET6-tCYC1 XI-3-pGAL1-5xluxO-yeGFP-tCPS1*	AAA112: pCfB6915 + NotI digested pAvA026	This study
**AAA198**	*MAT*-a *ura3 his3 leu2 trp1 XII-5-pPGK1-AaPKS3_co-tCYC pFAS1::pTEF1_luxO X-2-pTDH3-CepI-tCYC p-Cas9*	AAA134: pCfB6910 + NotI digested pAvA068	This study
**AAA200**	*MAT*-a *ura3 his3 leu2 trp1 XII-5-pPGK1-AaPKS3_co-tCYC pFAS1::pTEF1_luxO X-4-pPGK1-luxR_gen1-tADH1*	AAA134: pCfB3042 + NotI digested pAvA139	This study
**AAA202**	*MAT*-a *ura3 his3 leu2 trp1 XII-5-pPGK1-AaPKS3_co-tCYC pFAS1::pTEF1_luxO X-2-pTDH3-CepI-tCYC pCas9 VIII-1-tADH1-SAM2-pTEF1--pPGK1-MET6-tCYC1 X-4-pPGK1-luxR_gen1-tADH1*	AAA198: pCfB9340 + NotI digested155, pCfB6912 + NotI digested pAvA139	This study
**AAA203**	*MAT*-a *ura3 his3 leu2 trp1 XI-3-pTEF_luxO-eGFP-tCPS1 X-4-pPGK1-luxR_gen1-tADH1*	AAA095: pCfB3042 + NotI digested pAvA139	This study
**AAA206**	*MAT*-a *ura3 his3 leu2 trp1 XII-5-pPGK1-AaPKS3_co-tCYC pFAS1::pTEF1_LuxO VIII-1-tADH1-SAM2-pTEF1--pPGK1-MET6-tCYC1*	AAA198: pCfB9340 + NotI digested155	This study
**ACA001**	*MAT*-α *URA3 his3 LEU2 TRP1 p-Cas9*	CEN.PK110-10C: pTAJAK-161	This study
**ACA002**	*MAT*-α *URA3 his3 LEU2 TRP1 XI-3-pTEF1-amdS-tCPS1 p-Cas9*	ACA001: pCfB3045 + NotI digested pAvA085	This study
**ACA004**	*MAT*-α *URA3 his3 LEU2 TRP1 XI-3-pGAL1_core-5xluxO-amdS-tCPS1 p-Cas9*	ACA001: pCfB3045 + NotI digested pAvA087	This study
**ACA007**	*MAT*-α *URA3 his3 LEU2 TRP1 XI-3-pGAL1_core-5xluxO-amdS-yeGFP-tCPS1 p-Cas9*	ACA001: pCfB3045 + NotI digested pAvA061	This study
**ACA008**	*MAT*-α *URA3 his3 LEU2 TRP1 XI-3-pGAL1_core-5xluxO-amdS-mKate2-tCPS1 p-Cas9*	ACA001: pCfB3045 + NotI digested pAvA063	This study
**ACA013**	*MAT*-α *URA3 his3 LEU2 TRP1 XI-3-pTEF1-amdS-yeGFP-tCPS1 p-Cas9*	ACA001: pCfB3045 + NotI digested pAvA055	This study
**ACA015**	*MAT*-α *URA3 his3 LEU2 TRP1 XI-3-pGAL1_core-5xluxO-amdS-tCPS1 XII-5-pGAL1_core-5xluxO-yeGFP-tCPS1 p-Cas9*	ACA007: pCfB3050 + NotI digested pAvA117	This study
**ACA019**	*MAT*-α *URA3 his3 LEU2 TRP1 XI-3-pGAL1_core-5xluxO-amdS-tCPS1*	ACA004: pCfB3042 + NotI digested pAvA028	
**ACA030L**	*MAT*-α *URA3 his3 LEU2 TRP1 XI-3-pGAL1_core-5xluxO-amdS-tCPS1 XII-5-pGAL1_core-5xluxO-yeGFP-tCPS1 library X-4-pPGK1-GAL4_AD-NLS-luxR-tADH1, p-Cas9*	ACA015: pCfB3042 + X-4-p*PGK1* + *GAL4*_AD-*luxR* (library) + t*ADH1*-X-4	This study

Table S5: **Codon optimized DNA sequences of synthases and sequence of p*TEF1*_luxO_105.** The luxO operator sequence is underlined.

CepI	ATGCAAACTTTTGTTCATGAAGAAGGTAGATTGCCACATGAATTGGCTGCTGATTTGGGTAGATATAGAAGAAGAGT<br>TTTTGTTGAACAATTGGGTTGGGCTTTGCCATCTGCTAATGAATCTTTTGAAAGAGATCAATTTGATAGAGATGATAC<br>TGTTTATGTTTTTGCTAGAAATGCTGATGGTGATATGTGTGGTTGTGCTAGATTGTTGCCAACTACTAGACCATATTTG<br>TTGAAATCTTTGTTTGCTGATTTGGTTGCTGAAGATATGCCATTGCCACAATCTGCTGCTGTTTGGGAATTGTCTAGA<br>TTTGCTGCTACTGATGATGAAGGTGGTCCAGGTAATGCTGAATGGGCTGTTAGACCAATGTTGGCTGCTGTTGTTG<br>AATGTGCTGCTCAATTGGGTGCTAGACAATTGATTGGTGTTACTTTTGCTTCTATGGAAAGATTGTTTAGAAGAATTG<br>GTATTCATGCTCATAGAGCTGGTCCACCAAAACAAGTTGATGGTAGATTGGTTGTTGCTTGTTGGATTGATATTGATC<br>CACAAACTTTTGCTGCTTTGGGTATTGAACCAGGTCAAGCTGCTAGACAAGCTATTGCTGCTTCATAA
EsaI	ATGTTGGAGTTGTTCGATGTCTCCTACGAAGAATTGCAAACTACTAGATCTGAAGAGTTG<br>TACAAGCTGAGGAAAAAGACCTTCTCTGATAGATTAGGTTGGGAAGTTATTTGCTCCCAA<br>GGTATGGAATCTGATGAATTTGATGGTCCAGGTACTAGGTACATTTTGGGTATTTGTGAA<br>GGTCAATTGGTTTGCTCTGTTAGGTTCACTTCTTTGGATAGACCAAACATGATTACCCAT<br>ACCTTCCAACACTGTTTCTCTGATGTTACTTTGCCAGCTTATGGCACTGAATCTTCTAGA<br>TTCTTTGTTGATAAGGCTAGAGCTAGAGCTTTGTTGGGTGAACATTATCCAATCTCTCAA<br>GTTTTGTTCTTGGCCATGGTTAATTGGGCTCAAAACAATGCTTACGGTAACATCTACACC<br>ATCGTTTCAAGAGCCATGTTGAAGATTTTGACAAGATCTGGTTGGCAGATCAAGGTTATC<br>AAAGAAGCTTTCTTGACCGAGAAAGAGAGGATCTATTTGTTGACATTGCCAGCTGGTCAA<br>GATGACAAACAACAATTAGGTGGTGATGTTGTTTCTAGAACTGGTTGTCCACCAGTTGCT<br>GTTACTACTTGGCCATTGACTTTACCAGTTTAA
LasI	ATGATCGTCCAAATCGGTAGAAGAGAAGAGTTCGACAAAAAGTTGTTGGGTGAAATGCAT<br>AAGTTGAGAGCCCAGGTTTTCAAAGAAAGAAAAGGTTGGGATGTTTCCGTCATCGACGAA<br>ATGGAAATTGATGGTTATGATGCCTTGTCTCCCTACTACATGTTGATTCAAGAAGATACT<br>CCAGAGGCTCAAGTTTTTGGTTGTTGGAGAATTTTTGATACCACCGGTCCATATATGCTG<br>AAGAATACTTTCCCAGAACTGTTGCATGGTAAAGAAGCACCTTGTTCTCCACATATTTGG<br>GAATTGTCTAGATTCGCCATTAACTCTGGTCAAAAAGGTTCTTTGGGTTTCTCTGATTGC<br>ACTTTGGAAGCTATGAGAGCTTTGGCTAGATACTCCTTGCAAAACGATATTCAAACCTTG<br>GTTACTGTTACCACTGTTGGTGTTGAAAAGATGATGATTAGAGCCGGTTTGGATGTCTCT<br>AGATTTGGTCCACATTTGAAGATCGGTATTGAAAGAGCTGTTGCCTTGAGAATTGAATTG<br>AACGCTAAAACCCAAATCGCCTTGTATGGTGGTGTTTTGGTTGAACAAAGATTGGCCGTT<br>TCTTAA
LuxI	ATGACCATCATGATCAAGAAGTCCGATTTCTTGGCTATTCCATCTGAAGAGTACAAGGGT<br>ATCTTGTCCTTGAGATACCAAGTGTTCAAGCAAAGATTGGAATGGGACTTAGTTGTCGAA<br>AACAACTTGGAATCCGATGAGTACGATAATTCTAACGCTGAGTATATCTACGCCTGTGAT<br>GATACTGAAAACGTTTCTGGTTGTTGGAGATTATTGCCAACTACTGGTGATTACATGCTG<br>AAGTCTGTTTTTCCAGAACTGTTGGGTCAACAATCTGCTCCAAAAGATCCAAACATCGTC<br>GAATTGTCTAGATTCGCTGTTGGTAAGAACTCCTCCAAGATTAACAATTCCGCTTCCGAA<br>ATTACCATGAAGTTGTTCGAAGCCATCTACAAACATGCTGTTTCTCAAGGTATTACCGAA<br>TACGTTACTGTTACCTCTACCGCCATTGAAAGATTCTTGAAGAGAATCAAGGTTCCATGC<br>CATAGAATCGGTGACAAAGAAATTCATGTTTTGGGTGACACCAAGTCCGTTGTTTTGTCT<br>ATGCCAATCAACGAACAATTCAAGAAGGCCGTTTTGAACTAA
p*TEF1*<br>_luxO<br>-105	GCACACACCATAGCTTCAAAATGTTTCTACTCCTTTTTTACTCTTCCAGATTTTCTCGGACTCCGCGCATCGCCGT<br>ACCACTTCAAAACACCCAAGCACAGCATACTAAATTTCCCCTCTTTCTTCCTCTAGGGTGTCGTTAATTACCCGTA<br>CTAAAGGTTTGGAAAAGAAAAAAGAGACCGCCTCGTTTCTTTTTCTTCGTCGAAAAAGGCAATAAAAATTTTTATCA<br>CGTTTCTTTTTCTTGAAAATTTTTTTTTTTGATTTTTTTCTCTTTCGATGACCTCCCATTGATATTTAAGTTAATAAACGGT<br>CTTCACCTGTAGGATCGTACAGGTAATTTCTCAAGTTTCAGTTTCATTTTTCTTGTTCTATTACAACTTTTTTTACTTCTT<br>GCTCATTAGAAAGAAAGCATAGCAATCTAATCTAAGTTTTAATTACAA
p*TEF1*<br>_luxO<br>*-105	GCACACACCATAGCTTCAAAATGTTTCTACTCCTTTTTTACTCTTCCAGATTTTCTCGGACTCCGCGCATCGCCGT<br>ACCACTTCAAAACACCCAAGCACAGCATACTAAATTTCCCCTCTTTCTTCCTCTAGGGTGTCGTTAATTACCCGTA<br>CTAAAGGTTTGGAAAAGAAAAAAGAGACCGCCTCGTTTCTTTTTCTTCGTCGAAAAAGGCAATAAAAATTTTTATCA<br>CGTTTCTTTTTCTTGAAAATTTTTTTTTTTGATTTTTTTCTCTTTCGATGACCTCCCATTGATATTTAAGTTAATAAACGGT<br>CTTCACCTGTCCGATCGGACAGTATAATTTCTCAAGTTTCAGTTTCATTTTTCTTGTTCTATTACAACTTTTTTTACTTC<br>TTGCTCATTAGAAAGAAAGCATAGCAATCTAATCTAAGTTTTAATTACAA

Table S6: MRM transitions for homoserine lactones quantified by LC-MS/MS.

Analyte	Retention Time [min]	Q1 [*m/z*]	Q3 [*m/z*]	Fragmentor [V]	CE [V]
Aloesone [M+H]+	1.86	233.1	191.0 Qt	92	17
		233.1	151.0	92	37
		233.1	77.0	92	49
C4-HSL [M+H]+	0.85	172.1	102.1 Qt	53	5
		172.1	154.1	53	5
		172.1	71.0	53	9
C6-HSL [M+H]+	1.91	200.1	102.1 Qt	63	5
		200.1	182.1	63	5
		200.1	71.1	63	13
C6-oxo-HSL [M+H]+	1.29	214.1	102.1 Qt	58	5
		214.1	186.1	58	5
		214.1	74.0	58	21
C8-HSL [M+H]+	1.9	228.1	102.1 Qt	63	5
		228.1	210.1	63	5
		228.1	74.0	63	21
C8-oxo-HSL [M+H]+	2.6	242.1	102.1 Qt	63	9
		242.1	141.1	63	9
		242.1	184.1	63	9
C10-HSL [M+H]+	3.13	256.1	102.1 Qt	78	9
		256.1	238.2	78	5
		256.1	155.1	78	5
C12-oxo-HSL [M+H]+	3.24	298.1	102.1 Qt	82	9
		298.1	240.1	82	9
		298.1	155.1	82	13

Qt = quantifier ion, additional transitions were used for identification. Q = quadrupole. CE = collision energy.

References

1. Abramson, J. *et al.* Accurate structure prediction of biomolecular interactions with AlphaFold 3. *Nature* **630**, 493–500 (2024).

2. Lengger, B. *et al.* Serotonin G Protein-Coupled Receptor-Based Biosensing Modalities in Yeast. *ACS Sens* **7**, 1323–1335 (2022).

3. Jessop-Fabre, M. M. *et al.* EasyClone-MarkerFree: A vector toolkit for marker-less integration of genes into Saccharomyces cerevisiae via CRISPR-Cas9. *Biotechnol J* **11**, 1110–1117 (2016).

4. Zhang, J. *et al.* A microbial supply chain for production of the anti-cancer drug vinblastine. *Nature* **609**, 341–347 (2022).

5. Babaei, M. *et al.* Expansion of EasyClone-MarkerFree toolkit for Saccharomyces cerevisiae genome with new integration sites. *FEMS Yeast Res* **21**, foab027 (2021).

6. Tominaga, M., Nozaki, K., Umeno, D., Ishii, J. & Kondo, A. Robust and flexible platform for directed evolution of yeast genetic switches. *Nat Commun* **12**, 1846 (2021).

7. Grant, P. K. *et al.* Orthogonal intercellular signaling for programmed spatial behavior. *Mol Syst Biol* **12**, 849 (2016).

8. Shong, J. & Collins, C. H. Engineering the esaR Promoter for Tunable Quorum Sensing-Dependent Gene Expression. *ACS Synth Biol* **2**, 568–575 (2013).

9. Entian, K.-D. & Kötter, P. 25 yeast genetic strain and plasmid collections. *Methods in microbiology* **36**, 629–666 (2007).
