# Supplementary material for: Engineering N-acyl-homoserine lactone-based quorum-sensing circuit for dynamic regulatory control in Saccharomyces cerevisiae
Source: Commun Biol. 2025 Nov 27;8:1793. doi: 10.1038/s42003-025-09163-9 (PMC12717128; doi:10.1038/s42003-025-09163-9)
Supplement: Supplementary file 2 — Description of Additional Supplementary Files [file 42003_2025_9163_MOESM2_ESM.docx]

**Description of Additional Supplementary Files**

File name - Supplementary Data 1

File description - source data behind the graphs in the main text of the paper

File name - Supplementary Data 2

File description - source data behind the graphs in the supplementary information
