# Supplementary material for: Engineering N-acyl-homoserine lactone-based quorum-sensing circuit for dynamic regulatory control in Saccharomyces cerevisiae
Source: Commun Biol. 2025 Nov 27;8:1793. doi: 10.1038/s42003-025-09163-9 (PMC12717128; doi:10.1038/s42003-025-09163-9)
Supplement: Supplementary file 5 — Reporting Summary [file 42003_2025_9163_MOESM5_ESM.pdf]

nature portfolio

Corresponding author(s): Aafke C.A. van Aalst, Emil Damgaard Jensen

Last updated by author(s): Sep 22, 2025

# Reporting Summary

Nature Portfolio wishes to improve the reproducibility of the work that we publish. This form provides structure for consistency and transparency in reporting. For further information on Nature Portfolio policies, see our Editorial Policies and the Editorial Policy Checklist.

## Statistics

For all statistical analyses, confirm that the following items are present in the figure legend, table legend, main text, or Methods section.

| n/a | Confirmed | |
|---|---|---|
| ☐ | ☒ | The exact sample size (*n*) for each experimental group/condition, given as a discrete number and unit of measurement |
| ☐ | ☒ | A statement on whether measurements were taken from distinct samples or whether the same sample was measured repeatedly |
| ☐ | ☒ | The statistical test(s) used AND whether they are one- or two-sided<br>*Only common tests should be described solely by name; describe more complex techniques in the Methods section.* |
| ☐ | ☒ | A description of all covariates tested |
| ☐ | ☒ | A description of any assumptions or corrections, such as tests of normality and adjustment for multiple comparisons |
| ☐ | ☒ | A full description of the statistical parameters including central tendency (e.g. means) or other basic estimates (e.g. regression coefficient) AND variation (e.g. standard deviation) or associated estimates of uncertainty (e.g. confidence intervals) |
| ☒ | ☐ | For null hypothesis testing, the test statistic (e.g. *F*, *t*, *r*) with confidence intervals, effect sizes, degrees of freedom and *P* value noted<br>*Give P values as exact values whenever suitable.* |
| ☒ | ☐ | For Bayesian analysis, information on the choice of priors and Markov chain Monte Carlo settings |
| ☒ | ☐ | For hierarchical and complex designs, identification of the appropriate level for tests and full reporting of outcomes |
| ☒ | ☐ | Estimates of effect sizes (e.g. Cohen's *d*, Pearson's *r*), indicating how they were calculated |

*Our web collection on statistics for biologists contains articles on many of the points above.*

## Software and code

Policy information about availability of computer code

| | |
|---|---|
| Data collection | NovoExpress Software (Agilent) and BD FACSDiva Software (BD Biosciences) |
| Data analysis | GraphPad Prism was used for statistical analyses. BD FlowJo software was used to analyze the flow cytometry data. Pymol software was used to generate protein structures |

For manuscripts utilizing custom algorithms or software that are central to the research but not yet described in published literature, software must be made available to editors and reviewers. We strongly encourage code deposition in a community repository (e.g. GitHub). See the Nature Portfolio guidelines for submitting code & software for further information.

## Data

Policy information about availability of data

All manuscripts must include a data availability statement. This statement should provide the following information, where applicable:

- Accession codes, unique identifiers, or web links for publicly available datasets
- A description of any restrictions on data availability
- For clinical datasets or third party data, please ensure that the statement adheres to our policy

All data shown in figures are available in the Source data provided with this paper. There are no restrictions on data availability.

## Research involving human participants, their data, or biological material

Policy information about studies with human participants or human data. See also policy information about sex, gender (identity/presentation), and sexual orientation and race, ethnicity and racism.

| | |
|---|---|
| Reporting on sex and gender | n/a |
| Reporting on race, ethnicity, or other socially relevant groupings | n/a |
| Population characteristics | n/a |
| Recruitment | n/a |
| Ethics oversight | n/a |

Note that full information on the approval of the study protocol must also be provided in the manuscript.

# Field-specific reporting

Please select the one below that is the best fit for your research. If you are not sure, read the appropriate sections before making your selection.

☒ Life sciences ☐ Behavioural & social sciences ☐ Ecological, evolutionary & environmental sciences

For a reference copy of the document with all sections, see nature.com/documents/nr-reporting-summary-flat.pdf

# Life sciences study design

All studies must disclose on these points even when the disclosure is negative.

| | |
|---|---|
| Sample size | Sample sizes for each experiment are indicated in the figure legends. For flow cytometry analysis 2-3 biological replicates were used and 20,000 events were recorded. SInce data was normally distributed and obtained results were highly reproducible, we considered the sample sizes sufficient. For biolector fedbatch experiment we used 4-5 biological replicates and 3 technical replicates to conclude consistent and significant differences between the strains. |
| Data exclusions | No data was excluded |
| Replication | All attempts at replication were successful. Internal controls (control yeast strains or control conditions) were always included in each experiment and performed similarly for each independent experiment. Reproducibility of yeast-based experiments were ensured by using fresh -70C glycerol culture aliquots to start pre-cultures. |
| Randomization | Single colonies from transformation plates and from library population were randomly picked. |
| Blinding | Blinding was done for LC-MS data acquisition for the first synthase screening experiments. For yeast growth experiments, no blinding was performed since it involved bulk samples of rationally engineered used yeast cells. |

# Reporting for specific materials, systems and methods

We require information from authors about some types of materials, experimental systems and methods used in many studies. Here, indicate whether each material, system or method listed is relevant to your study. If you are not sure if a list item applies to your research, read the appropriate section before selecting a response.

## Materials & experimental systems

| n/a | Involved in the study | |
|---|---|---|
| ☒ | ☐ | Antibodies |
| ☒ | ☐ | Eukaryotic cell lines |
| ☒ | ☐ | Palaeontology and archaeology |
| ☒ | ☐ | Animals and other organisms |
| ☒ | ☐ | Clinical data |
| ☒ | ☐ | Dual use research of concern |
| ☒ | ☐ | Plants |

## Methods

| n/a | Involved in the study | |
|---|---|---|
| ☒ | ☐ | ChIP-seq |
| ☐ | ☒ | Flow cytometry |
| ☒ | ☐ | MRI-based neuroimaging |

## Plants

Seed stocks

*Report on the source of all seed stocks or other plant material used. If applicable, state the seed stock centre and catalogue number. If plant specimens were collected from the field, describe the collection location, date and sampling procedures.*

Novel plant genotypes

*Describe the methods by which all novel plant genotypes were produced. This includes those generated by transgenic approaches, gene editing, chemical/radiation-based mutagenesis and hybridization. For transgenic lines, describe the transformation method, the number of independent lines analyzed and the generation upon which experiments were performed. For gene-edited lines, describe the editor used, the endogenous sequence targeted for editing, the targeting guide RNA sequence (if applicable) and how the editor was applied.*

Authentication

*Describe any authentication procedures for each seed stock used or novel genotype generated. Describe any experiments used to assess the effect of a mutation and, where applicable, how potential secondary effects (e.g. second site T-DNA insertions, mosiacism, off-target gene editing) were examined.*

## Flow Cytometry

### Plots

Confirm that:

☒ The axis labels state the marker and fluorochrome used (e.g. CD4-FITC).

☒ The axis scales are clearly visible. Include numbers along axes only for bottom left plot of group (a 'group' is an analysis of identical markers).

☒ All plots are contour plots with outliers or pseudocolor plots.

☒ A numerical value for number of cells or percentage (with statistics) is provided.

### Methodology

Sample preparation

A 1 mL aliquot of glycerol -70°C freezer stock was used to inoculate 50 mL of SC medium or a single colony from the screened library was inoculated into 200 µL of SC medium and grown for 16 hours. For analysis of ligand-dependent activation, the culture was diluted 1:20 in 200 µL SC medium ± inducer in 96-deep-well culture plates and grown for 6 hours at 30°C. For analysis of ligand-dependent repression, the culture was diluted 1:50 and grown for 24 hours prior to being diluted 1:20 in 200 µL SC medium ± inducer in 96-deep-well culture plates and grown for 6 hours. Cells were washed and diluted 1:4 in PBS prior to analysis by flow cytometer

Instrument

NovoCyte QuanteonTM (Agilent)

Software

FlowJo

Cell population abundance

20,000 cells with a threshold of FSC-H 150,000

Gating strategy

FSC-A was plotted against FSC-H to gate for singlets events. Gated events were used to determine the median fluorescence (FITC-H) of the population.

☒ Tick this box to confirm that a figure exemplifying the gating strategy is provided in the Supplementary Information.
